# Intestinal Parasitic Infections among Intellectually Disabled Individuals in Bandar Abbas County, Southern Iran

**DOI:** 10.1155/2022/8406636

**Published:** 2022-07-12

**Authors:** Abbas Pakmehr, Mostafa Omidian, Habibollah Turki, Mohammad Fararouei, Bahador Sarkari

**Affiliations:** ^1^Department of Parasitology and Mycology, School of Medicine, Shiraz University of Medical Sciences, Shiraz, Iran; ^2^Infectious and Tropical Diseases Research Center, Hormozgan Health Institute, Hormozgan University of Medical Sciences, Bandar Abbas, Iran; ^3^HIV/AIDS Research Center, Department of Epidemiology, School of Health, Shiraz University of Medical Sciences, Shiraz, Iran; ^4^Basic Sciences in Infectious Diseases Research Center, Shiraz University of Medical Sciences, Shiraz, Iran

## Abstract

Intellectually disabled individuals are more prone to parasitic infections due to their unusual behaviors, immune and nutrient deficiencies, and living conditions. The current study is aimed at evaluating the prevalence of intestinal parasites in institutionalized intellectually disabled individuals in Bandar Abbas County in the south of Iran. Subjects of the study were 119 individuals, living in an intellectually disabled individual care center. Demographic features of the subjects including age, sex, intellectual disability type, and duration of their stay in the center were recorded. A stool sample was taken from each subject and evaluated by direct wet mount and formalin-ethyl-acetate concentration methods. Samples were also examined, using trichrome and modified acid-fast permanent staining. The mean age of the subjects was 27.6 (±2.24), ranging from 4 to 60 years old. Of the 119 participants, 55 (46.2%) were male, and 64 cases (53.8%) were female. Overall, 31 individuals (26.1%, 95% CI: 18.4-34.9) were found to be infected with at least one type of intestinal parasite. *Blastocystis hominis*, as the most common detected parasite, was detected in 13 (10.1%), *Entamoeba coli* in 12 (10.1%), *Giardia lamblia* in 5 (4.2%), *Cryptosporidium* in 2 (1.7%), *Iodamoeba butchlii* in 2 (1.7%), and *Endolimax nana* in 1 (0.8%) of participants. Three cases had coinfection with *Blastocystis hominis* and *Entamoeba coli*, one case was infected with *Blastocystis hominis* and *Giardia lamblia*, and one case was coinfected with *Giardia lamblia* and *Entamoeba coli*. There were no statistically significant associations between intestinal parasitic infection and gender, age, type of intellectual disability, or duration of stay in the care center (*P* > 0.05). The findings of the present study indicate a relatively high prevalence of parasitic infections in people with intellectual disabilities in Bandar Abbas, southern Iran. Noteworthy is the high prevalence of *Blastocyst* and also the presence of *Cryptosporidium* infection in these people. Periodic treatment of these people and improvement of their maintenance conditions can be considered for the prevention and control of intestinal parasitic infection in these people.

## 1. Introduction

Parasitic infections are global health problems, especially in developing countries. Parasitic infections affect 3.5 billion people worldwide, and 450 million of them, mostly children, suffer from intestinal parasites [1]. Intestinal parasites cause gastrointestinal problems, iron deficiency anemia, malnutrition, weight loss, abdominal pain, malabsorption, or even cognitive disability [2]. Parasitic diseases cause 16 million deaths annually. Preschool- and school-aged children are more susceptible to intestinal parasites than adults [3]. The prevalence of intestinal parasites in a particular area depends on various factors including social conditions, poverty, overcrowded populations, lack of access to health facilities, poor nutrition, and poor health care [4].

People with intellectual disabilities are at risk of parasitic infections due to their poor self-care skills and personal hygiene and lack of health education and performing high-risk behaviors [5–10]. Underlying diseases in these people such as immune and nutrient deficiencies, weakness, and constipation along with unusual behavior such as geophagia, nail-biting, confronting human waste, and poor hygiene make them more vulnerable to infectious diseases including parasitic infections. Moreover, person-to-person transmission is an important route for transmission of parasitic infections in densely populated areas, where people with intellectual disabilities usually live.

The current study is aimed at evaluating the prevalence of intestinal parasites in institutionalized intellectually disabled individuals in Bandar Abbas County in the south of Iran.

## 2. Materials and Methods

### 2.1. Study Population and Sample Collection

The study was conducted in a rehabilitation center for intellectual-disabled individuals in Bandar Abbas city capital of Hormozgan province, southern Iran. Bandar Abbas is situated in a flat area, 9 meters above sea level. The annual rainfall in this region is about 171 mm, the maximum temperature in summer reaches 49°C, and the minimum temperature drops to 5°C in the winter.

Stool specimens from 117 individuals with intellectual disabilities were collected and preserved in SAF stock solution in a mouthed screw-capped container, from February to March 2021. Each container was properly labeled and transferred to the parasitology laboratory at Shiraz University of Medical Sciences. Along with sampling, a consent form and questionnaire containing demographic information and some possible risk factors for intestinal parasitic infection were collected from parents of the subjects or rehabilitation center officials. The study was approved by the Ethics Committee of the Shiraz University of Medical Sciences. Confidentiality of the details of the participants was assured.

### 2.2. Stool Examination

All stool specimens were evaluated with the direct wet mount and formalin-ethyl-acetate concentration methods. Also, with the help of human serum, thinly spread stool samples on glass slides were prepared and stained with a trichrome permanent stain. Moreover, diarrheic samples were evaluated with a modified acid-fast staining method for the detection of coccidian parasites. An agar plate culture was used for the detection of *Strongyloides stercoralis* larvae.

### 2.3. Statistical Analysis

For the statistical analysis of collected data, SPSS software (version 20) was used. The prevalence of parasitic infections was determined by descriptive statistics. Chi-squared test was used to determine any association between the variables and the positivity of intestinal parasites. The level of significance was set at 5%.

## 3. Results

The mean age of the subjects was 27.6 (±2.24), ranging from 4 to 60 years old. Of 119 participants, 55 (46.2%) were male, and 64 (53.8%) cases were female. The age group of 31-40 years included most of the subjects (27.2%). Overall, 31 individuals (26%), were infected with at least one type of intestinal parasite, and five of them had coinfection with two different intestinal parasites.

*Blastocystis hominis*, as the most common detected parasite, was detected in 13 (10.1%), *Entamoeba coli* in 12 (10.1%), *Giardia lamblia* in 5 (4.2%), *Cryptosporidium* in 2 (1.7%), *Iodamoeba butchlii* in 2 (1.7%), and *Endolimax nana* in 1 (0.8%) case. Three cases had coinfection with *Blastocystis hominis* and *Entamoeba coli*, one case was infected with *Blastocystis hominis* and *Giardia lamblia*, and one case was infected with *Giardia lamblia* and *Entamoeba coli*.  Table 1 shows the prevalence of intestinal parasitic infection among intellectually disabled individuals in Bandar Abbas County, Southern Iran, based on different detection methods.

There were no significant associations between intestinal parasitic infection in the studied subjects and gender, age, type of intellectual disability, or duration of stay in the care center (*P* > 0.05) (Tables 2–6). A statistically significant association was found between the type of parasite and the macroscopic form of the fecal sample where both *Cryptosporidium*-infected cases had diarrheic stool form. In this study, no helminthic infection was found in any of the studied samples.

## 4. Discussion

Intestinal parasites are an important cause of gastrointestinal disorders. The main symptoms of these infections are anorexia, diarrhea, weight loss, abdominal pain, nausea, and anemia. People with intellectual disabilities due to poor health, having certain behaviors such as nail-biting, and geophagia, and also living in crowded environments, are more at risk of acquiring parasitic infections. The current study is aimed at evaluating the rate of intestinal parasitic infections in a care center for people with intellectual disabilities in southern Iran. The results of the study showed that 26% of these people are infected with at least one intestinal parasite.

*Blastocystis hominis* was the most common detected parasite followed by *Entamoeba coli*, *Giardia lamblia*, *Cryptosporidium*, *Iodamoeba butchlii*, and *Endolimax nana*. *Blastocystis* is considered a common protozoan infection in humans in Iran [11–16]. Results of a recent systematic review and meta-analysis revealed that *Entamoeba coli*, *Blastocystis* spp., and *Giardia lamblia* are the most prevalent protozoan parasite among intellectually disabled individuals in Iran [11].

In previous years, two other studies have been conducted on intellectually disabled individuals in Bandar Abbas, where the current study has been undertaken, and both reported a higher prevalence of intestinal parasites in these people [7, 17]. In those studies, the prevalence of *Strongyloides stercoralis* was reported to be 17.3% and 20.6%, while none of the subjects in our study, despite using the formalin-ethyl-acetate concentration and agar plate culture methods, were found to be infected with this helminth [7, 17]. The reason for the substantial difference in the prevalence of infection in these people in the present study in comparison with the previous two studies can be due to improving the care of these people, the awareness of staff working in that center about parasitic infection and, most importantly, periodic antiparasitic treatment of the subjects based on the findings and recommendations of the previous studies. Apart from *Strongyloides stercoralis* infection, other parasitic infections in the present in comparison with previous studies have the same variation but less prevalence rate.

In the current study, no association was found between the age of participants and the frequency of parasitic infection. This is not surprising as all people living in this center are equally exposed to parasitic infections and have similar risky behaviors for parasitic diseases. Nevertheless, in some studies, including the study of Tappeh et al. on 225 individuals with different disability grades and Sharif's study on intellectually disabled children in northern Iran, such association has been reported [18, 19]. It is worth mentioning that in our study, unlike Sharif and Tappe study, all of our subjects had high-grade of disability that makes these people, regardless of their age, unteachable, and have behaviors that expose them to parasitic infections. Also, in our study, there was no statistically significant association between the prevalence of intestinal parasites and gender which is consistent with the previous studies [7, 9, 18–20].

*Giardia lamblia* was one of the most prevalent parasites seen in the studied subjects in the current study. It is noteworthy that all *Giardia*-infected individuals in the present study were cyst-passers. Cyst passes are mainly asymptomatic, and they can easily transmit the infection to people who are close to them.

In our study, with the help of acid-fast permanent staining, infection with *Cryptosporidium* was detected in two cases, both diarrheic. This parasite is exclusively important because it can cause important gastrointestinal disorders, especially in children. *Blastocystis hominis* was the most prevalent protozoan infection in our study. Although there is no consensus among experts as to whether this parasite is pathogenic or not, there are reports of a significant association between *Blastocystis* infection and irritable bowel syndrome in humans [13, 15, 21, 22]. Also, the role of *Blastocystis* in causing inflammatory bowel disease and variations in the intestinal microbial flora is proposed [23]. In recent years, a relatively high prevalence of *Giardia* and *Blastocystis* infection has been reported in people with various disorders, including IBS [24, 25]. High prevalence of these two protozoan infections among intellectually disabled individuals in the current study might be linked to the IBS disorder.

The incidence of intestinal helminthiases in Iran has drastically decreased in the last decades [26, 27]. The prevalence of ascariasis and strongyloidiasis lies just between 0.1% and 0.3%, and there appears that less than 1% of the Iranian population is now infected with hookworms [26]. In the present study, no case of helminth infection was seen in the subjects. Periodic administration of antihelminth drugs to intellectually disabled individuals following the recommendations of researchers in previous studies, and the very low prevalence of helminth infections in different regions of Iran can be attributed to the absence of helminthic infection in these people in the current study.

## 5. Conclusion

The findings of the present study indicate a relatively high prevalence of parasitic infections in people with intellectual disabilities in Bandar Abbas, southern Iran. Noteworthy is the high prevalence of *Blastocyst* and also the presence of *Cryptosporidium* infection in these people. Periodic treatment of these people and improvement of their maintenance conditions is recommended for the prevention and control of intestinal parasitic infection in this group of people.

## Figures and Tables

**Table 1:** Detected parasites among intellectually disabled individuals in Bandar Abbas County, Southern Iran, by different detection methods.

Intestinal parasite	Wet mount technique	Formalin-ethyl-acetate concentration	Trichrome and modified acid-fast staining	Total (%)
*Blastocystis hominis*	12	13	13	13 (10.1)
*Entamoeba coli*	11	12	12	12 (10.1)
*Giardia lambelia*	4	5	5	5 (4.2)
*Cryptosporidium*	0	0	2	2 (1.7)
*Iodamoeba butschlii*	2	2	2	2 (1.7)
*Endolimax nana*	0	1	1	1 (0.8)

**Table 2:** Frequency of parasitic infections, based on gender, among intellectually disabled individuals in Bandar Abbas County, Southern Iran.

Gender	Frequency (%)	Positive for intestinal parasites (%)	Prevalence (95% CI)	*P* value
Female	64 (53.8)	15 (48.4)	23.4 (13.8-35.7)	0.534
Male	55 (46.2)	16 (51.6)	29.1 (17.6-42.9)	
Total	119 (100.0)	31 (100)	26.1 (18.4-34.9)	

**Table 3:** Frequency of intestinal parasitic infections based on age, among intellectually disabled individuals in Bandar Abbas County, Southern Iran.

Age group (yr.)	Frequency (%)	Positive for intestinal parasites (%)	Prevalence 95% CI	*P* value
≤10	9 (7.6)	3 (9.7)	33.3 (7.5-70.0)	0.437
11-20	30 (25.2)	11 (35.5)	36.7 (19.9-56.1)	
21-30	31 (26.1)	7 (22.6)	22.6 (9.6-41.1)	
31-40	33 (27.7)	8 (25.8)	24.2 (11.1-42.3)	
>40	16 (13.4)	2 (6.5)	12.5 (1.6-38.4)	
Total	119 (100.0)	31 (100)	26.1 (18.4-34.9)	

**Table 4:** Frequency of parasitic infections, based on the duration of stay in the care center, among intellectually disabled individuals in Bandar Abbas County, Southern Iran.

Duration of presence in the center (yr.)	Frequency (%)	Positive for intestinal parasites (%)	Prevalence 95% CI	*P* value
≤5	44 (37.0)	10 (32.3)	22.7 (11.5-37.8)	0.314
6-10	23 (19.3)	10 (32.3)	43.5 (23.2-65.5)	
11-15	25 (21.0)	6 (19.4)	24.0 (9.4-45.1)	
16-20	10 (8.4)	2 (6.5)	20.0 (2.5-55.6)	
>20	17 (14.3)	3 (9.7)	17.7 (3.8-43.4)	
Total	119 (100.0)	31 (100)	26.1 (18.4-34.9)	

**Table 5:** Frequency of parasitic infections, based on the intellectually disabled type, among intellectually disabled individuals in Bandar Abbas County, Southern Iran.

Disability type	Frequency (%)	Positive for intestinal parasites (%)	Prevalence 95% CI	*P* value
Severe	76 (63.9)	19 (38.7)	25.0 (15.8-36.3)	0.729
Profound	43 (36.1)	12 (61.3)	27.9 (15.3-43.7)	
Total	119 (100.0)	31 (100)	26.1 (18.4-34.9)	

**Table 6:** Results of logistic regression measuring association between the study variables and parasitic infection.

	OR	95% CI		*P* value
		Lower	Upper	
Gender				
Male	Ref	—	—	.696
Female	.837	0.344	2.038	
Duration				
≤5	Ref			.521
6-10	.924	0.165	5.160	
11-15	2.498	0.458	13.619	—
16-20	1.103	0.193	6.284	—
>20	1.048	0.128	8.568	—
Age				
≤10	Ref	—	—	.636
11-20	4.247	0.423	42.687	
21-30	3.719	0.622	22.258	—
31-40	2.129	0.351	12.915	—
>40	2.403	0.430	13.421	—
Retardation				
Severe	Ref	—	—	.965
Profound	.979	0.370	2.588	

## Data Availability

The datasets generated and analyzed during the current study are available in the tables. The quantitative data used to support the findings of this study are available from the corresponding author upon request.
